# Depletion of Shine-Dalgarno Sequences Within Bacterial Coding Regions Is Expression Dependent

**DOI:** 10.1534/g3.116.032227

**Published:** 2016-09-07

**Authors:** Chuyue Yang, Adam J. Hockenberry, Michael C. Jewett, Luís A. N. Amaral

**Affiliations:** * Department of Chemical and Biological Engineering, Northwestern University, Evanston, Illinois 60208; † Interdisciplinary Program in Biological Sciences, Northwestern University, Evanston, Illinois 60208; ‡ Northwestern Institute on Complex Systems, Northwestern University, Evanston, Illinois 60208; § Chemistry of Life Processes Institute, Northwestern University, Evanston, Illinois 60208; ** Department of Physics and Astronomy, Northwestern University, Evanston, Illinois 60208

**Keywords:** translation initiation, gene expression, growth regulation

## Abstract

Efficient and accurate protein synthesis is crucial for organismal survival in competitive environments. Translation efficiency (the number of proteins translated from a single mRNA in a given time period) is the combined result of differential translation initiation, elongation, and termination rates. Previous research identified the Shine-Dalgarno (SD) sequence as a modulator of translation initiation in bacterial genes, while codon usage biases are frequently implicated as a primary determinant of elongation rate variation. Recent studies have suggested that SD sequences within coding sequences may negatively affect translation elongation speed, but this claim remains controversial. Here, we present a metric to quantify the prevalence of SD sequences in coding regions. We analyze hundreds of bacterial genomes and find that the coding sequences of highly expressed genes systematically contain fewer SD sequences than expected, yielding a robust correlation between the normalized occurrence of SD sites and protein abundances across a range of bacterial taxa. We further show that depletion of SD sequences within ribosomal protein genes is correlated with organismal growth rates, supporting the hypothesis of strong selection against the presence of these sequences in coding regions and suggesting their association with translation efficiency in bacteria.

Translation of mRNA to protein consumes a vast amount of cellular resources, particularly in rapidly growing unicellular organisms (Dekel and Alon 2005; Wagner 2005; Shachrai *et al.* 2010). Many researchers have hypothesized that efficient (*i.e.*, fast and accurate) translation is highly advantageous and should therefore leave a recognizable signature on the genome (Sharp *et al.* 2005; Stoletzki and Eyre-Walker 2007; Drummond and Wilke 2008; Supek *et al.* 2010; Vieira-Silva and Rocha 2010; Botzman and Margalit 2011).

For decades, researchers have focused on understanding the link between tRNA concentration and translation rates of cognate codons, under the assumption that ribosomal dwell-time on a particular codon is partially determined by diffusion limited tRNA binding and competition between near-cognates (Ikemura 1981; dos Reis *et al.* 2004; Rocha 2004). Indeed, multiple lines of evidence strongly support this hypothesis in a multitude of different organisms (Tuller *et al.* 2010).

Recently, ribosome profiling (a technique that maps transcriptome-wide ribosome occupancy) has been applied to study whether different codons show variation in translation rates, but researchers have come to conflicting conclusions, even when using the same dataset (Li *et al.* 2012; Dana and Tuller 2014; Gardin *et al.* 2014; Hussmann *et al.* 2015; Weinberg *et al.* 2016). One of the most startling findings to emerge from ribosome profiling experiments is the striking degree of heterogeneity in ribosome occupancy across mRNAs, which is punctuated by large peaks suggestive of “pausing” or “stalling” (Ingolia *et al.* 2009; Li *et al.* 2012; Schrader *et al.* 2014). These pauses, in contrast to known stalling sequences, are orders of magnitude larger than what is expected from basal translation rate variations due to tRNA concentrations, and may instead result from nascent peptide interactions within the ribosomal exit tunnel (such as poly-proline sequences), ribosomal queuing, or *trans*-interactions between mRNA and ribosomes (Li *et al.* 2012; Charneski and Hurst 2013; Shah *et al.* 2013; Woolstenhulme *et al.* 2015; Weinberg *et al.* 2016).

Using ribosome profiling, Li *et al.* (2012) showed that, in bacteria, translational pauses were significantly associated with sequence binding between the anti-Shine-Dalgarno (aSD) sequence of the 16S ribosomal-RNA and the translating message. This binding interaction is important during the process of translation initiation, where the ribosome binds to the 5′ untranslated region (5′-UTR) to facilitate start codon recognition (Figure 1A). However, the occurrence of these “Shine-Dalgarno” (SD) sequences within coding sequences had not been previously associated with translational pausing (Shine and Dalgarno 1974; Salis *et al.* 2009). SD sequence-mediated pauses have now been documented for several bacterial species and independent ribosomal profiling datasets (Li *et al.* 2012; Liu *et al.* 2013; Schrader *et al.* 2014). Studies have built on these results by showing SD-associated pauses *in vitro*, negative effects of SD sequences on protein production in engineered sequences, enhanced solubility of recombinant proteins via rational insertion of SD sequences at protein domain boundaries, and enrichment of SD sequences following transmembrane domains of natural sequences (Agashe *et al.* 2013; Chen *et al.* 2014; Chevance *et al.* 2014; Fluman *et al.* 2014; Vasquez *et al.* 2015).

**Figure 1:**
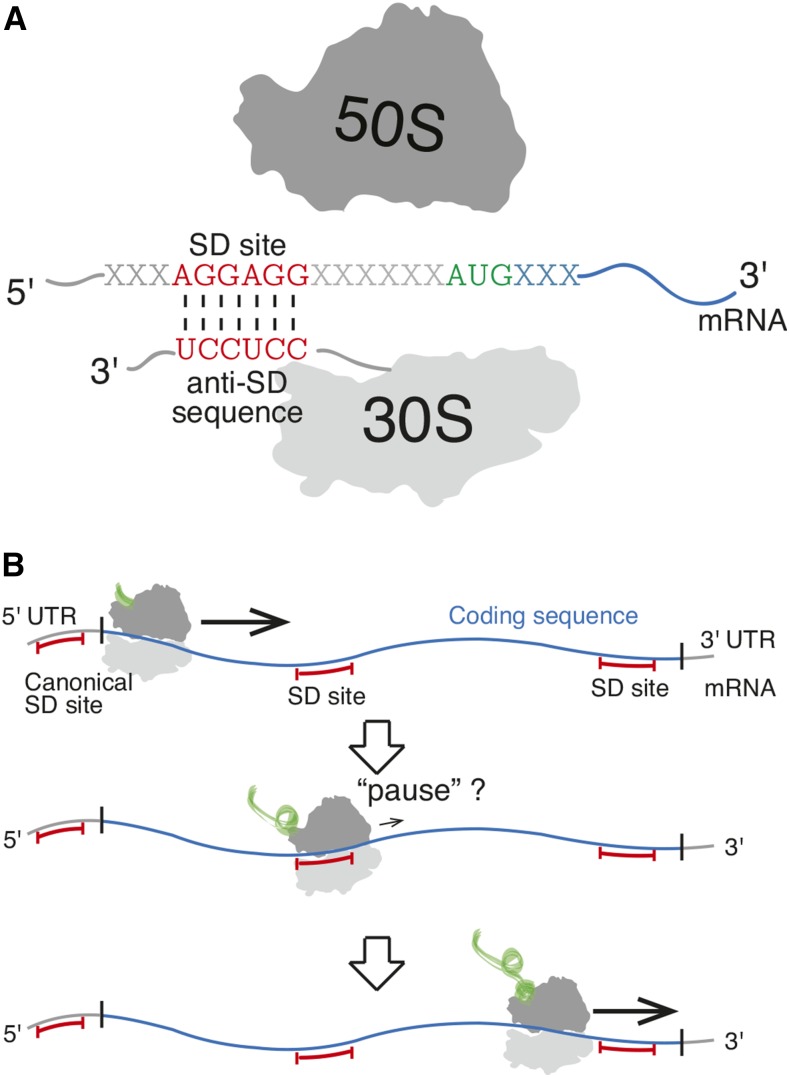
The possible dual impacts of Shine-Dalgarno (SD) sequences on protein synthesis. (A) SD sequences in the 5′ untranslated region (UTR) of mRNA (messenger RNA) are known to facilitate translation initiation in bacteria via binding to the anti-SD sequence on the 3′ tail of the 16S ribosomal RNA. (B) Recent research suggests that SD sequences within coding sequences may regulate the rate of translation elongation.

By contrast, recent results have questioned whether the observed SD-associated pauses are actually an experimental artifact resulting from the ribosome profiling protocol, specifically the differential sizes of sequencing fragments (O’Connor *et al.* 2013; Mohammad *et al.* 2016). Indeed, the existence of SD-mediated pauses has not been confirmed using several other experimental methods (Borg and Ehrenberg 2015; Chadani *et al.* 2016; Mohammad *et al.* 2016). Thus, remains unclear what role, if any, SD sequences within protein coding genes have in modulating translation speed (Figure 1B).

Even though the usage and diversity of SD sequences within the 5′-UTR has been analyzed extensively at the genome-scale (Ma *et al.* 2002; Starmer *et al.* 2006; Nakagawa *et al.* 2010), the occurrence pattern of these important sequence motifs within the coding sequences of diverse species has been largely neglected [though see Itzkovitz *et al.* (2010) for an exception]. Thus, open questions remain as to whether SD sequences are indeed systematically depleted within coding sequences from diverse species and, if so, whether the depletion follows any particular pattern that may provide clues to the evolutionary significance of these sequences.

In order to answer these open questions, we sought to characterize the general occurrence of SD sequences within protein coding genes across a range of bacterial species of known phylogeny. We first present a metric to characterize single mRNA sequences according to their estimated sequence binding propensity with the ribosomal aSD sequence. Using this metric, we show that depletion of SD sequences in coding regions is a hallmark of bacterial genes and that, within a given species, the degree of this depletion is inversely correlated with measured gene expression levels. Finally, we show that variation in SD sequence depletion between different genomes is related to the minimal known doubling time of individual species, suggesting that depletion of SD sequences is driven by evolutionary pressure for greater translation efficiency.

## Materials and Methods

### Codon-shuffled null model

We randomly generated null model genomes that preserve codon usage and primary amino acid sequence at the gene level. For each gene, we constructed a list of all codons used in the original sequence. Given the primary amino acid sequence of the gene, we then randomly selected a codon from the pool of available synonymous codons for that particular amino acid without replacement. The start and stop codons are not affected by this process and thus remain fixed during the shuffling process. We repeated this procedure for every gene within a given genome in order to create one instance of a randomized genome for null model comparison. For statistical comparison using Monte Carlo hypothesis testing, we created 1000 randomized genomes in this manner. Using our metric, we calculated the mean and SD in these randomized genomes for each organism, and then calculated a *z*-score for the real genome along with the resulting *p* value, which we report in the main text.

### aSD hybridization

We predicted thermodynamic interactions between the aSD sequence and each six-nucleotide-long sequence using the RNA cofold method of the ViennaRNA Package 2.0 with default parameters (Gruber *et al.* 2008). For this study, we have chosen to use the canonical core aSD sequence of 5′-CCUCCU-3′ for all species, owing to the fact that this core sequence is nearly universally conserved. Further, the 3′-tail of 16s rRNAs is slightly variable and poorly annotated (Nakagawa *et al.* 2010; Lim *et al.* 2012), making it difficult to empirically determine the precise aSD sequence for each individual species.

### Pax-Db data collection

We collected the complete bacterial dataset from the Protein Abundance Across Organisms Database (Pax-Db) in August 2015 (Wang *et al.* 2015). This resource contains protein abundance measurements for 26 different bacteria. When multiple datasets were available for a particular organism, we chose the “Integrated” dataset, which is the result of Pax-Db curators integrating the various protein abundance data sources based on coverage and quality. The full set of data that we analyzed for each species is available upon request.

### Growth-rate dataset and phylogenetic relatedness

We obtained growth rate measurements (minimum doubling time, measured in hr) from Vieira-Silva and Rocha (2010). For each species in their data table, we matched the name of the species provided in the original data source to the species name in a local copy of the NCBI GenBank complete genome sequences. This resulted in 187 matches for bacteria (Archaeal species, which were provided in the original dataset, were ignored for the purposes of this study). Within each of these bacterial genomes, we relied on annotations in the GenBank files to find ribosomal proteins by searching the “product” field for “ribosomal subunit,” or perturbations thereof. Full data, including GenBank files for all relevant organisms and ribosomal protein “locus_tags” used in this study, are available upon request.

To construct a phylogenetic tree from these species, we extracted the 23S and 16S gene sequences using RNAmmer-1.2 (Lagesen *et al.* 2007). When multiple sequences were available for a given genome, we randomly chose one of each for alignment. We then individually aligned 23S and 16S sequences using MUSCLE (Edgar 2004). Finally, we concatenated the 16S and 23S alignments for each organism and constructed a maximum likelihood (ML) tree using RAxML with a partitioned analysis that separately fit rate models for the 16S and 23S sequences. We used a 5′-GTRGAMMA-3′ evolutionary model with 100 rapid bootstrap searches and 20 ML searches and selected the best fitting ML tree. (Stamatakis 2014).

### Regression analyses

With one exception noted below, all statistical analyses were performed using the SciPy (version: 0.16.0) and StatsModels (version: 0.6.1) packages in Python.

To control for phylogenetic effects in our growth rates regression analysis, we used the PGLS function from the “caper” package in R, choosing the optimal λ value to transform our input tree via maximum likelihood search.

### Data availability

The authors state that all data and code necessary for confirming the conclusions presented in the article are available as Supplementary Material (Table S3).

## Results

### Quantifying the occurrence of SD sequences within coding sequences

We first counted the number of occurrences of the canonical SD motif (5′-AGGAGG-3′) within the coding sequences of the 187 bacterial species compiled by Vieira-Silva and Rocha (2010). For each genome, we compared the number of SD sequences found within coding sequences to the number expected by chance using a codon-shuffled null model to control for codon usage bias within each gene (see *Materials and Methods*). We found that 175 out of 187 genomes contained fewer canonical SD sequences in their coding sequences than expected by chance (154 were significant at p<0.0001, Monte Carlo hypothesis testing, Figure 3A).

**Figure 2:**
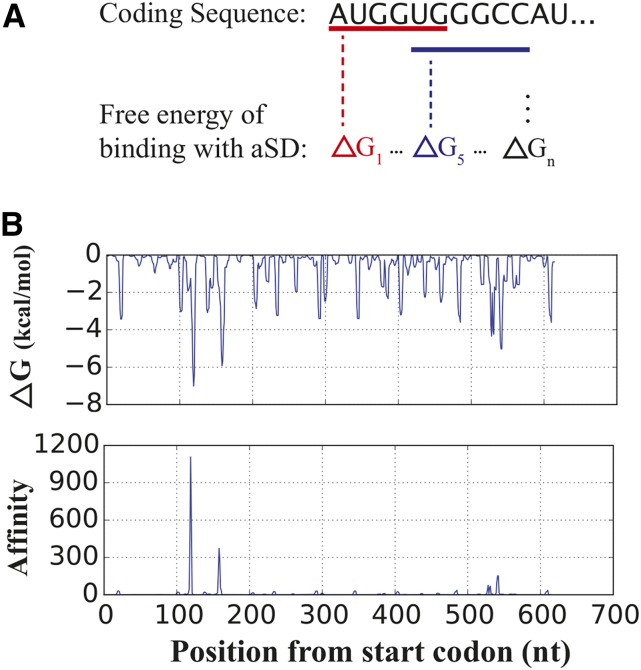
Quantifying aSD sequence binding within coding regions. (A) We estimate the free energy of binding for each hexamer within a gene to the core aSD (anti-Shine-Dalgarno) sequence (5′-CCUCCU-3′). (B) Free energy (top) and affinity (bottom) profiles for a typical *E. coli* gene (b3055). The affinity profile amplifies the contribution from strongly binding regions within the gene. nt, nucleotides.

However, single or multiple base mismatches to the canonical SD sequence are frequently assumed to be functional in translation initiation, and the strength of aSD sequence binding to different hexamer sequences spans a range of values. To quantify the occurrence of SD sequences on a per-gene basis in a manner that encapsulates the full breadth of this heterogeneity, we estimated the free energy of binding between the aSD sequence and each hexamer within the coding region of each mRNA (Figure 2A, see *Materials and Methods* for details). Since the free energy of binding (ΔG) at a particular site is proportional to the logarithm of the ratio of the association and dissociation rate constants of binding, we define the affinity *A* of a hexamer $\left\{n_{1}\ldots n_{6}\right\}$ to the aSD sequence as:

$$A_{\left\{n_{1}\ldots n_{6}\right\}}\equiv \exp\left(\left|\text{Δ}G_{\left\{n_{1},\ldots ,n_{6}\right\}}\right|\right)$$ (1)

We define the aSD binding score *S* of a gene as:

$$S_{\mathrm{gene}}\equiv \log\bar{A},$$ (2)

where $\bar{A}$ is the average affinity over a gene’s coding sequence (Figure 2B). The transformations involved in the definition of *S* aim to lessen the contribution of weak-binding interactions while amplifying the contributions from the strongest aSD binding sequences.

**Figure 3:**
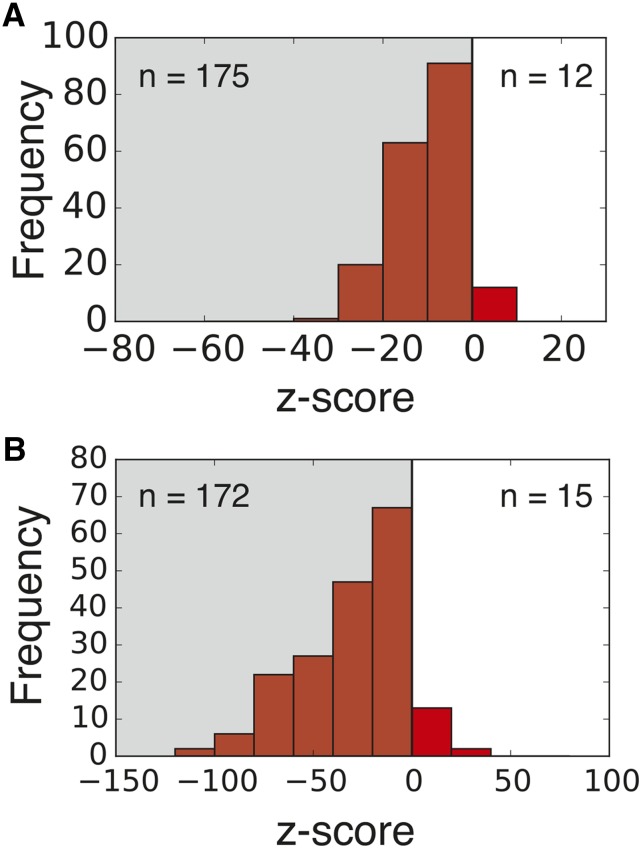
Depletion of SD occurrence in genomes compared to expectation from 1000 randomly generated genomes using our codon-shuffled null model. (A) the canonical SD (Shine-Dalgarno) sequence 5′-AGGAGG-3′ is depleted within coding sequences in most genomes (175 of 187). (B) The genome aSD (anti-SD) binding score $S_{\text{genome}}$ is lower for most organisms (172 of 187). Both distributions are centered significantly to the left of 0, showing that the majority of organisms avoid SD sequences according to both metrics.

We calculated $S_{\mathrm{gene}}$ for each of the coding sequences of 187 bacterial species, and define genome aSD binding score $S_{\text{genome}}={\bar{S}}_{\mathrm{gene}}.$ We again compared this empirical value to the expected value for a given genome based off a codon-shuffled null model and found that, similar to the previous analysis, 172 out of 187 genomes had average aSD binding scores lower than expected by chance (167 were significant at p<0.0001, Monte Carlo hypothesis test, Figure 3B). These results demonstrate that genomes contain significantly fewer SD sequences than would be expected from gene-specific codon usage biases and amino acid sequences.

### The occurrence of SD sequences in coding regions correlates negatively with Escherichia coli gene expression data

**Figure 4:**
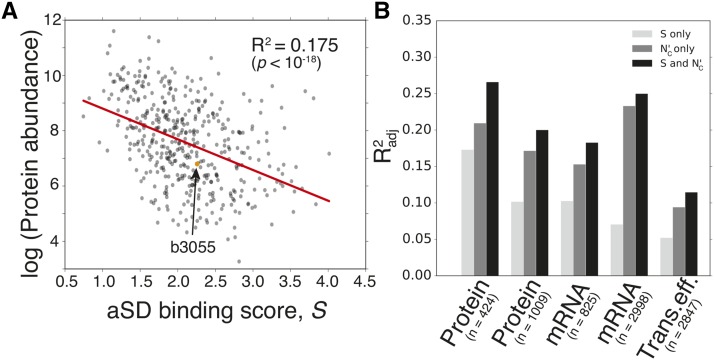
aSD binding scores negatively correlate with gene expression in *E. coli*. (A) An example dataset showing negative correlation between protein abundance and aSD (anti- Shine-Dalgarno) binding scores for individual *E. coli* genes ($R_{\mathrm{adj}}^{2}=0.175,$ $p<10^{-18}$). Specifically, coding sequences containing fewer SD sequence motifs have higher protein abundances. (B) Multivariate regression shows that expression changes cannot be fully explained by codon usage bias, and that additional predictive power is offered by $S_{\mathrm{gene}}.$ We chose five datasets that provide independent measurements of mRNA (messenger RNA), protein, and translation efficiency levels in order to test the robustness of our findings (Lu *et al.* 2007; Taniguchi *et al.* 2010; Shiroguchi *et al.* 2012; Li *et al.* 2014).

$S_{\mathrm{gene}}$ allows us to test whether variation in aSD sequence binding between different genes correlates with gene-level features such as expression level. We obtained five genome-scale expression datasets for *E. coli* to ensure the robustness of our results (Table S1) and correlated the gene expression measurements against the calculated aSD binding score for each gene (Figure 4A) (Lu *et al.* 2007; Taniguchi *et al.* 2010; Shiroguchi *et al.* 2012; Li *et al.* 2014). We observed a highly significant negative relationship in all datasets, indicating that the coding sequences of highly expressed genes contain fewer SD sequences ($p<10^{-18},$ for all cases) (Figure 4, A and B).

A number of different factors are known to influence protein abundances, including start codon choice, mRNA structural accessibility, and SD sequence usage at translation initiation sites (Guimaraes *et al.* 2014). Here, we wish to focus on the elongation phase of translational control to determine what, if any, additional predictive power is conferred by the effect of aSD sequence binding within coding sequences. Prior studies have established that the codon usage bias of individual genes is highly correlated with protein levels (Tuller *et al.* 2010). In order to investigate whether the observed correlation between $S_{\mathrm{gene}}$ and gene expression is driven solely by codon usage bias, we conducted multivariable linear regression using both *S* and an established method for quantifying codon usage bias to predict expression levels ($N_{c}^{\prime }$) (Novembre 2002). If *S* were solely a consequence of codon usage bias, the adjusted-$R^{2}$ ($R_{\mathrm{adj}}^{2}$) should decrease when *S* is included as an independent variable along with $N_{c}^{\prime }.$ On the contrary, we observe that the best model for all datasets includes both $N_{c}^{\prime }$ and *S* as predictors of expression (Figure 4B and Table S1). While the enhancement in predictive power is not additive, this is not uncommon when evaluating models with multiple covarying predictors.

### The occurrence of SD sequences within coding regions correlates negatively with protein abundances in diverse bacterial taxa

**Figure 5:**
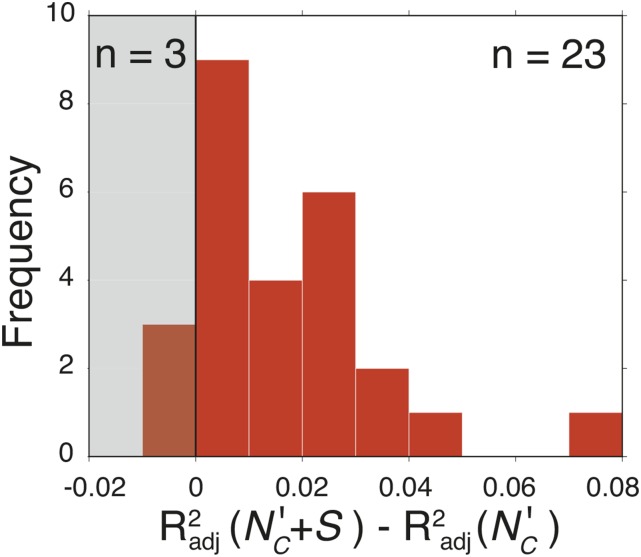
Shine-Dalgarno (SD) sequence depletion is correlated with protein abundances in a diverse set of bacterial taxa. Distribution of differences between the $R_{\mathrm{adj}}^{2}$ for models which do and do not contain the *S* score. For 23 of the 26 organisms, inclusion of aSD (anti-SD) binding score as an independent variable enhances predictive power. The full data table, including organism names and values, is available in Table S2.

To determine the generality of the previous finding, we expanded our analysis to 26 diverse bacteria for whom protein expression data were previously collected by Wang *et al.* (2015) (see *Materials and Methods*). For 19 out of 26 datasets, we observed that *S* was significantly negatively correlated (p<0.01) with protein abundances (Figure 5 and Table S2). As in the previous subsection, we also implemented a multivariate model to determine whether the observed correlation was solely a consequence of codon usage bias. For 23 out of 26 datasets we saw an improved $R_{\mathrm{adj}}^{2}$ value when $S_{\mathrm{gene}}$ is added as a predictor along with estimates of codon usage bias (Figure 5).

We further confirmed the observation that the more complex multivariate model resulted in a better fit to the data by using *AIC* and *BIC* to evaluate model fits. For 22 and 18 organisms, respectively, the multivariate model provided a better fit to the data than a linear model based on codon usage bias alone (Figure S1).

### Ribosomal protein coding sequences contain fewer SD sequences than other genes

To overcome the limited availability of bacterial protein expression datasets, we next investigated whether ribosomal protein coding sequences contain fewer SD sequences than other genes within a genome. Ribosomal proteins are essential for all organisms and they are generally expressed at high levels, making them some of the most likely genes to show selection for accurate and efficient translation.

**Figure 6:**
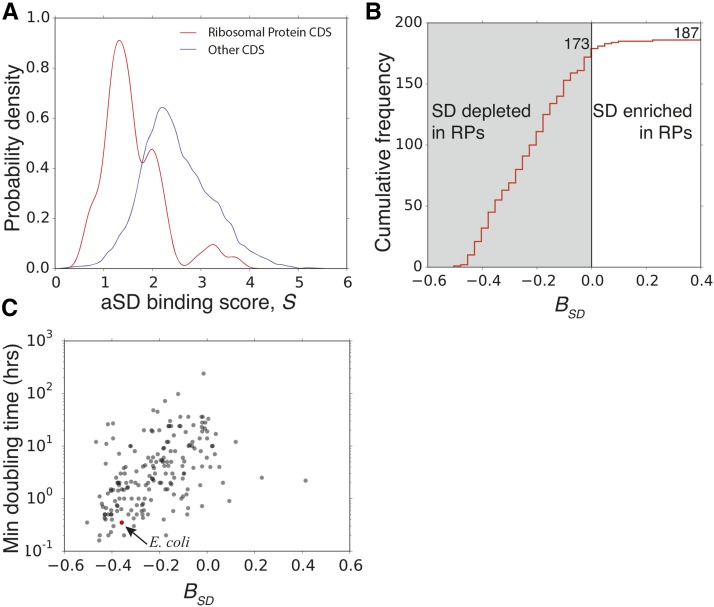
Depletion of SD (Shine-Dalgarno) sequences within ribosomal protein coding genes is widespread throughout the bacterial kingdom and associated with organismal growth. (A) Distribution of aSD (anti-SD) binding scores of ribosomal protein coding sequences in *E. coli*, compared to that of all other protein coding sequences. We characterize SD sequence usage bias in a genome with Equation (3). (B) Distribution of genome SD bias index for 187 bacteria genomes. Ribosomal proteins have significantly lower aSD binding scores, as compared to the rest of the genome, in the majority of bacterial species. (C) SD bias is correlated with minimum generation time in 187 organisms (Spearman-rank: ρ=0.530, $p<10^{-14}$). Depletion of internal-SD sequences in ribosomal protein genes is associated with faster growth. The full data table for this analysis, including organism names, growth rate, and *B* values, is provided as (Table S3).

In *E. coli*, we observed that aSD binding scores for the 58 ribosomal protein genes are significantly lower than that of all other genes (Figure 6A). To quantify the magnitude of this difference, we define the normalized SD bias within a genome, $B_{\mathrm{SD}},$ as:

$$B_{\mathrm{SD}}=\frac{{\bar{S}}_{\text{ribosome}\;\text{genes}}-{\bar{S}}_{\text{genome}}}{{\bar{S}}_{\text{genome}}}\times 100\%$$ (3)

where ${\bar{S}}_{\text{ribosome}\;\text{genes}}$ is the averaged $S_{\mathrm{gene}}$ for ribosomal protein coding genes, and ${\bar{S}}_{\text{genome}}$ is the averaged $S_{\mathrm{gene}}$ for all genes within a genome. When $B_{\mathrm{SD}}<0,$ ribosomal protein genes contain fewer SD sequences than would be expected based on the genome-wide average. We opt for this approach for two primary reasons. First, the *S* values of ribosomal protein coding genes themselves would be heavily influenced by the underlying genomic GC content. Normalizing to the genome-wide average should help to mitigate this effect. Second, research has shown that at higher growth rates, ribosomal protein genes make up an increasingly larger fraction of bacterial proteomes (Borkowski *et al.* 2016). Thus, relative differences in *S* between ribosomal protein coding genes and the genome as a whole should reflect the selective pressure for increased ribosomal protein production during periods of rapid growth.

Of the other 187 diverse bacteria spanning different genomic GC contents, growth environments, and growth rates, 173 have $B_{\mathrm{SD}}<0,$ suggesting that the vast majority of bacteria have a larger depletion of SD sequences in their ribosomal protein coding genes relative to the genome as a whole (Figure 6B). The systematic depletion of SD sequences in ribosomal protein coding sequences further suggests that these motifs negatively impact gene expression and/or cellular fitness in a wide diversity of bacteria.

Previous studies have shown that the relative codon usage bias of ribosomal genes compared to the rest of the genome is correlated with the minimum observed doubling time for particular species Vieira-Silva and Rocha (2010). This finding is mechanistically assumed to be a consequence of the fact that, at rapid growth rates, ribosomal proteins constitute an increasingly large fraction of the proteome; selection for translational accuracy or efficiency within these genes relative to the genome thus likely reflects the evolutionary history driven by growth rate demands. Therefore, we hypothesized that $B_{\mathrm{SD}}$ scores may also be related to the growth rate demands of individual species. Indeed, we found that $B_{\mathrm{SD}}$ is positively correlated with the minimum known doubling times of this set of 187 bacteria; fewer SD sequences within the ribosomal protein coding sequences relative to the genome is associated with faster maximal growth rates (Spearman-rank: ρ=0.530, $p<10^{-14}$) (Figure 6C). We further confirmed the robustness of this finding via phylogenetic generalized least squares regression (see *Materials and Methods*) (λ=0.978: $R_{\mathrm{adj}}^{2}=0.07,$ p=0.0002). This finding strongly suggests that SD motifs within coding sequences are detrimental to growth and reproduction, likely via negatively impacting translation.

## Discussion

Prior research into translation elongation has focused on codon usage as the primary means of modulating elongation speed, but researchers have recently proposed that aSD-mediated sequence interactions are a dominant source of translational pausing in bacteria (Gingold and Pilpel 2011; Li *et al.* 2012). If true, this finding has important consequences for our understanding of the basic mechanisms of translation as well as practical implications for coding sequence design for synthetic biology and biotechnological purposes. By quantifying the usage of SD sequences within coding sequences in a diverse set of bacterial taxa, we have shown a consistent trend whereby SD sequences within coding regions are systematically depleted. Specifically, this effect is strongest in the most highly expressed genes across a variety of genomes. We further show that the level of biased depletion of SD sequences is strongest in organisms capable of very rapid growth where selection for translation efficiency has previously been shown to produce a variety of genome-scale hallmarks (Vieira-Silva and Rocha 2010).

Recently, Diwan and Agashe (2016) published an elegant analysis of “internal-SD-like” sequence usage in prokaryotes. Our results largely confirm the major finding of this study, which showed internal-SD-like sequences are depleted in > 80% of the species analyzed. While their results found a number of species that were exceptions to this rule, we note that many of these exceptions are Archaea, whose translation initiation mechanisms remain elusive and are therefore excluded from our analysis. Further, our results build on these findings in important ways. By developing a metric of *S*, which is defined at the single-gene level, our analysis provides insight into within-genome variation and the selective pressures governing the usage of internal-SD sequences as it relates to gene expression costs. This within-genome analysis allows us to show that avoidance of SD sequences is highly related to the maximal growth rates of organisms using a method that controls for GC content variation, which Diwan and Agashe (2016) found to impose an important constraint on the appearance of internal-SD sequences. Our analysis does not focus on temperature or variation in internal-SD usage with regard to position within genes, but the thorough results of Diwan and Agashe (2016) likely hold within our dataset.

There are several possible limitations to our methodology that readers should be aware of when interpreting our findings. First, our study relies on an assumed aSD sequence of 5′-CCUCCU-3′ to calculate aSD binding strength scores for individual genes. It is possible, and evidence strongly suggests, that in particular lineages the aSD sequence may be slightly altered or extended compared to this canonical sequence (Lim *et al.* 2012). Therefore, we may be mischaracterizing the aSD sequence for several species in our dataset, or not encompassing the full breadth of possible sequence interactions. Future work can refine our findings to account for this aSD heterogeneity as more aSD sequences will be empirically determined, but we opt here for a conservative approach likely to be applicable for the majority of organisms in our dataset. Second, while our study relies on the precise definition of coding sequence bounds in existing genome annotations, prior research has shown that these annotations are likely spurious for up to ∼10% of annotated genes (Schrader *et al.* 2014; Nakahigashi *et al.* 2016). However, reliable N-terminal mapping is currently available for only a small fraction of bacterial genomes; until better computational models are developed to refine translational start site predictions, this will remain a limitation that adds noise to any computational genome-scale analysis, such as the one we perform here.

SD sequences may be avoided within coding sequences for several different, and nonmutually exclusive, reasons. These sequences may: (i) result in erroneous internal translation initiation leading to the production of truncated protein products; (ii) temporarily sequester ribosomes, thus limiting the number available for proper translation initiation; (iii) encourage translational frameshifting; or (iv) substantially slow down translation elongation (Devaraj and Fredrick 2010; Chu *et al.* 2011; Li *et al.* 2012; Whitaker *et al.* 2014). In all of these cases, we would expect SD sequences within coding sequences to be largely detrimental and thus avoided. In particular, given that the consequences of any of the above explanations is amplified by high mRNA copy numbers, avoidance of these SD sequences would also be expected to manifest particularly in the most highly expressed genes.

Although our results indicate that SD sequences are by and large detrimental, we also wish to clarify that some proportion of the SD sites within coding sequences may serve important functions. Owing to the compact nature of bacterial genomes, the translation initiation site of many genes within operons will occur within the 3′ terminus of the preceding coding sequence. Further, the presence of multiple translation initiation sites may serve a regulatory role for certain proteins, allowing for the production of distinct isoforms depending on the N-terminal sequence or controlling protein folding rates (Ozin *et al.* 2001; Fluman *et al.* 2014; Schrader *et al.* 2014; Vasquez *et al.* 2015).

One benefit of our large-scale analysis is that exceptions to the rules can point to interesting cases for further study. In Figure 5 we found three species where *S* did not appear to enhance predictions of protein abundance: *Mycoplasma pneumoniae*, *Shigella flexneri*, and *Leptospira interrogans*. Although none of these species are known to use noncanonical aSD sequences (Lim *et al.* 2012), all are pathogenic species, suggesting that a possible relationship may exist between ecological strategies, effective population size, and the selection against SD sequences. However, owing to the large number of pathogenic species in this dataset, this finding will require further detailed investigation. Additionally, several species analyzed in Figure 6 showed an enhancement of SD sequence usage within ribosomal proteins relative to the genome. Nearly all of these cases come from three distinct orders (phyla), pointing to likely mechanistic changes in the aSD interaction in particular clades: *Rickettsia* (Alphaproteobacteria), Mollicutes (Tenericutes) and Spirochaetes (Spirochaete) (both *M. pneumoniae* and *L. interrogans*, mentioned above, fall within one of these orders). Future ribosome profiling experiments on species from within these clades may provide clues on the evolution of the aSD sequence interaction.

The patterns that we observe provide significant insight into the debate surrounding the usage of SD sequences within protein coding genes. Moreover, our results are fully orthogonal to ribosome profiling-based conclusions. It is clear from this bioinformatic analysis that SD sequences are largely avoided across the bacterial kingdom, and that this avoidance is likely due to deleterious effects on translation. Thus, we conclude that even if SD-mediated elongation pausing is an artifact of the ribosomal profiling protocol, as suggested by Mohammad *et al.* (2016), care should be taken to avoid SD sequences when designing coding sequences for recombinant protein production applications.

## Supplementary Material

Supplemental Material
