# Supplementary material for: Depletion of Shine-Dalgarno Sequences Within Bacterial Coding Regions Is Expression Dependent
Source: G3 (Bethesda). 2016 Sep 7;6(11):3467–74. doi: 10.1534/g3.116.032227 (PMC5100845; doi:10.1534/g3.116.032227)
Supplement: Supplemental Material [file supp_6_11_3467__index.html]

Depletion of Shine-Dalgarno Sequences Within Bacterial Coding Regions Is Expression Dependent — Supplemental Material 

# Depletion of Shine-Dalgarno Sequences Within Bacterial Coding Regions Is Expression Dependent

## Supplemental Material for Yang *et al.*, 2016

**Files in this Data Supplement:**

- Figure S1 - Inclusion of the aSD binding strength score, *S*, to multivariate regression between protein abundance and codon usage bias (*N'C*) enhances total predictive power of multiple regression models as evidenced by lower (A) AIC and (B) BIC scores. (.pdf, 75 KB)
- Table S1 - Single and multivariable regression outputs for *E.coli* gene expression data, aSD binding score (*S*) and codon usage bias (*N'C*). (.pdf, 117 KB)
- Table S2 - Single and multivariable regression outputs between protein abundance, aSD binding score (*S*) and codon usage bias (*N'C*) in 26 bacteria.
- Table S3 - Primary data for genome-wide metrics and growth rate comparisons. Included are the species name in the original data table, the name and accession name of the matched genome in our database, doubling times, calculated Z-scores from Figure 3A&B, and the normalized SD bias from Figure 6C in the main text. (.csv, 26 KB)
- File S1 - Zip archive containing all the necessary Python code and processed data required to replicate findings from this paper, per instructions in the included readme.txt file. Replication from primary data is also possible with the given code, but requires the user to download the listed genome files. (.zip, 762 KB)
