# Supplementary material for: Depletion of Shine-Dalgarno Sequences Within Bacterial Coding Regions Is Expression Dependent
Source: G3 (Bethesda). 2016 Sep 7;6(11):3467–74. doi: 10.1534/g3.116.032227 (PMC5100845; doi:10.1534/g3.116.032227)
Supplement: Supplemental Material [file supp_g3.116.032227_FigureS1.pdf]

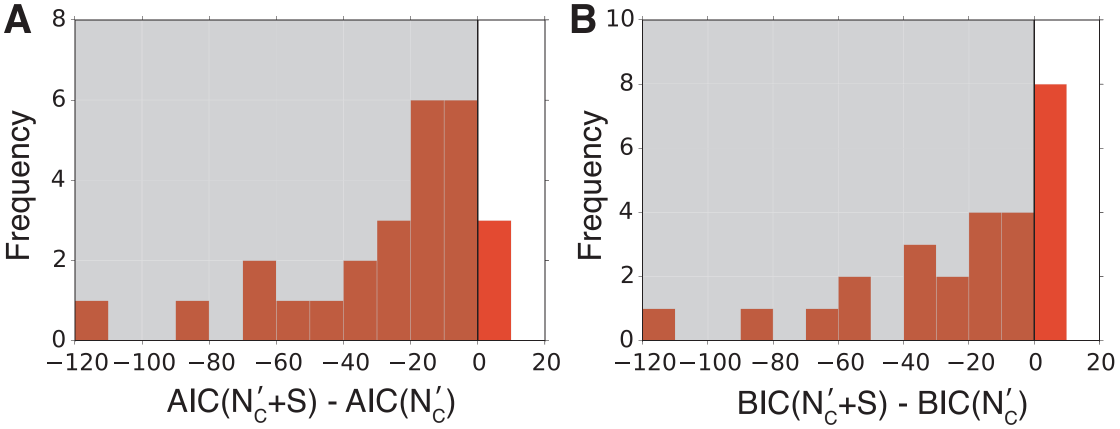

Figure S1: Inclusion of the aSD binding strength score, S, to multivariate regression between protein abundance and codon usage bias (N'_C) enhances total predictive power of mulitple regression models as evidenced by lower (A) AIC and (B) BIC scores.
