# Supplementary material for: Depletion of Shine-Dalgarno Sequences Within Bacterial Coding Regions Is Expression Dependent
Source: G3 (Bethesda). 2016 Sep 7;6(11):3467–74. doi: 10.1534/g3.116.032227 (PMC5100845; doi:10.1534/g3.116.032227)
Supplement: Supplemental Material [file supp_g3.116.032227_TableS1.pdf]

Table S1: Single and multivariable regression outputs for *E.coli* gene expression data, aSD binding score (S) and codon usage bias (N'_C).

Dataset	n	R^2_{SDonly}	p	slope	$R^2_{N'_C only}$	p	slope	R^2_{both}	p	$slope_{SD}$	$slope_{N'_C}$
Protein abundance (Lu *et al.* 2007).	424	0.173	2.179e-19	-1.111	0.209	1.441e-23	-4.975	0.266	1.971e-29	-0.713	-3.702
Mean protein abundance (Taniguchi *et al.* 2011)	1009	0.102	1.675e-25	-1.031	0.172	2.431e-43	-6.335	0.200	5.695e-50	-0.599	-5.223
mean mRNA level (Taniguchi *et al.* 2011)	825	0.103	2.308e-21	-0.827	0.153	8.828e-32	-4.663	0.183	3.321e-37	-0.495	-3.716
mRNA digital counts (Shiroguchi *et al.* 2012)	3002	0.071	7.657e-50	-0.595	0.233	2.097e-175	-5.615	0.250	1.805e-188	-0.305	-5.151
Translation efficiency (Li *et al.* 2014)	2877	0.052	1.427e-35	-0.280	0.094	4.521e-64	-1.908	0.115	3.339e-77	-0.185	-1.629
