# Supplementary material for: Depletion of Shine-Dalgarno Sequences Within Bacterial Coding Regions Is Expression Dependent
Source: G3 (Bethesda). 2016 Sep 7;6(11):3467–74. doi: 10.1534/g3.116.032227 (PMC5100845; doi:10.1534/g3.116.032227)
Supplement: Supplemental Material [file supp_g3.116.032227_TableS2.pdf]

Table S2: Single and multivariable regression outputs between protein abundance, aSD binding score (S) and codon usage bias (N'_C) in 26 bacteria.

Organism	n	R^2_{SD}	p	slope	$R^2_{N'_C}$	p	slope	R^2_{both}	p	$slope_{SD}$	$slope_{N'_C}$
Escherichia coli str. K-12 substr. MG1655	3736	0.0590	1.64E-051	-1.15	0.202	3.85E-185	-11.1	0.215	1.13E-197	-0.580	-10.2
Leptospira interrogans serovar Copenhageni str. Fiocruz L1-130	2392	0.00176	0.0224	0.130	0.0788	8.90E-045	-8.01	0.0784	1.57E-043	0.0128	-7.99
Campylobacter jejuni subsp. jejuni NCTC 11168 = ATCC 700819	769	0.00706	0.0112	-0.203	0.0718	2.48E-014	-5.05	0.0801	4.73E-015	-0.217	-5.09
Bartonella henselae Houston-1	1252	-0.000633	0.649	-0.0264	0.0943	5.63E-029	-6.18	0.0968	8.83E-029	-0.117	-6.34
Pseudomonas aeruginosa PAO1	2417	0.0565	1.30E-032	-0.564	0.0994	3.89E-057	-5.24	0.121	1.51E-068	-0.366	-4.44
Bacillus anthracis Sterne	1386	0.0672	5.99E-023	-0.720	0.208	1.72E-072	-6.63	0.225	1.27E-077	-0.377	-6.04
Staphylococcus aureus subsp. aureus Mu50	1683	0.0152	2.39E-007	-0.353	0.248	2.38E-106	-7.80	0.253	1.29E-107	-0.212	-7.68
Mycoplasma pneumoniae FH	381	-0.000267	0.344	0.102	0.00324	0.136	-1.29	0.00282	0.216	0.0985	-1.27
Streptococcus pyogenes M1 GAS	1284	0.0613	1.33E-019	-0.793	0.332	9.36E-115	-10.4	0.343	7.32E-118	-0.345	-9.89
Legionella pneumophila subsp. pneumophila str. Philadelphia 1	743	0.0366	9.02E-008	-0.442	0.0990	9.47E-019	-7.72	0.124	2.22E-022	-0.368	-7.29
Microcystis aeruginosa NIES-843	4090	0.000138	0.211	-0.0504	0.0902	2.92E-086	-6.74	0.0911	6.45E-086	-0.0848	-6.77
Shewanella oneidensis MR-1	1316	0.101	1.36E-032	-0.788	0.182	1.07E-059	-6.49	0.207	3.36E-067	-0.427	-5.42
Helicobacter pylori 26695	1410	0.00260	0.0309	0.166	0.00805	0.000433	-2.31	0.0101	0.000289	0.152	-2.24
Bacteroides thetaiotaomicron VPI-5482	768	0.112	9.15E-022	-1.00	0.115	2.77E-022	-6.68	0.162	1.82E-030	-0.713	-4.82
Desulfovibrio vulgaris str. Hildenborough	956	0.0755	2.98E-018	-0.645	0.160	2.82E-038	-6.53	0.174	1.00E-040	-0.311	-5.63
Lactococcus lactis subsp. lactis Il1403	1293	0.0898	1.83E-028	-0.812	0.246	1.38E-081	-7.23	0.286	1.39E-095	-0.554	-6.60
Neisseria meningitidis MC58	411	0.0109	0.0194	-0.198	0.00755	0.0430	-1.20	0.0112	0.0370	-0.151	-0.708
Deinococcus deserti VCD115	1300	0.0717	5.21E-023	-0.672	0.139	2.24E-044	-6.55	0.164	1.80E-051	-0.418	-5.62
Mycobacterium tuberculosis H37Rv	3320	0.00546	1.19E-005	0.275	0.0295	1.27E-023	-5.31	0.0338	6.09E-026	0.246	-5.21
Shigella flexneri 2a str. 301	1648	0.0156	2.09E-007	-0.470	0.391	1.85E-179	-11.1	0.390	7.10E-178	0.0253	-11.1
Bacillus subtilis subsp. subtilis str. 168	3914	0.0106	5.90E-011	-0.259	0.138	6.91E-129	-7.89	0.143	2.44E-132	-0.176	-7.76
Acidithiobacillus ferrooxidans ATCC 23270	1302	0.00655	0.00201	-0.158	0.0915	3.49E-029	-4.59	0.0942	4.54E-029	-0.107	-4.51
Listeria monocytogenes EGD-e	469	0.0545	1.88E-007	-0.703	0.172	3.57E-021	-6.19	0.193	6.76E-023	-0.457	-5.69
Salmonella enterica subsp. enterica serovar Typhimurium str. LT2	2561	0.0936	8.04E-057	-0.812	0.143	3.65E-088	-6.11	0.183	3.50E-113	-0.556	-5.07
Yersinia pestis CO92	1197	0.0498	3.46E-015	-0.640	0.0450	7.45E-014	-4.39	0.0692	9.21E-020	-0.483	-3.13
Synechocystis sp. PCC 6803	1183	0.0770	1.46E-022	-0.622	0.0294	1.70E-009	-3.29	0.101	1.73E-028	-0.602	-2.99
