# Supplementary figures and images for: Depletion of Shine-Dalgarno Sequences Within Bacterial Coding Regions Is Expression Dependent
Source: G3 (Bethesda). 2016 Sep 7;6(11):3467–74. doi: 10.1534/g3.116.032227 (PMC5100845; doi:10.1534/g3.116.032227)

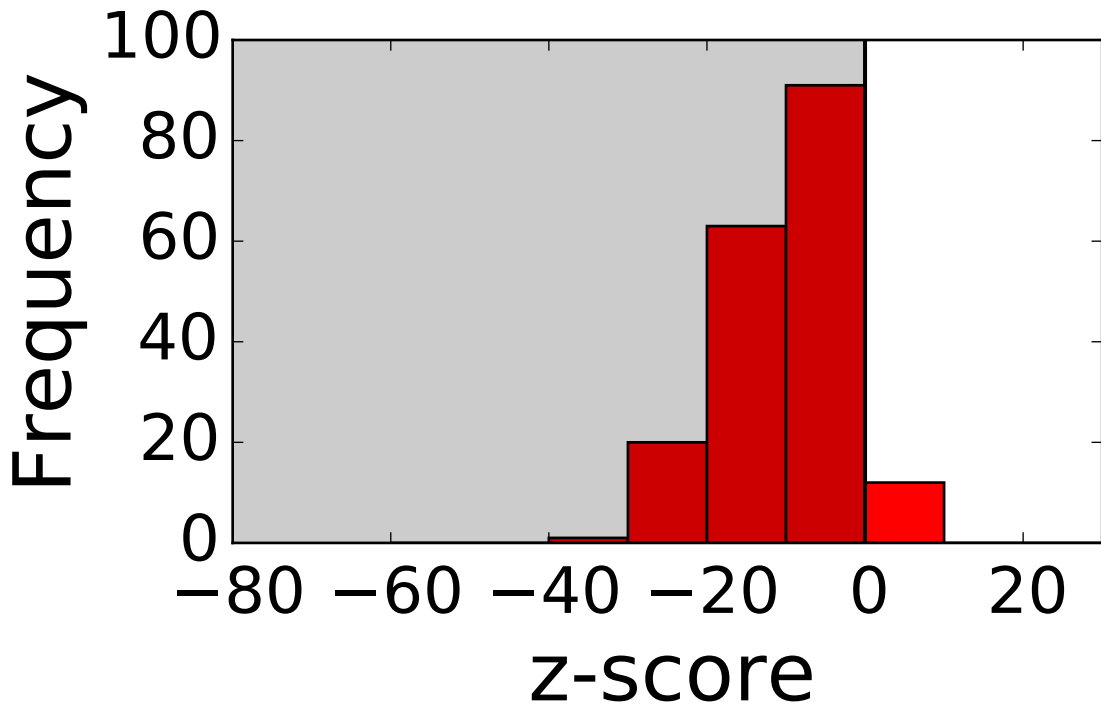

Supplement: Supplemental Material [file supp_g3.116.032227_FileS1.zip › SD_Submission 3/Results/Figures/3A-zscore-histogram-AGGAGG.pdf]

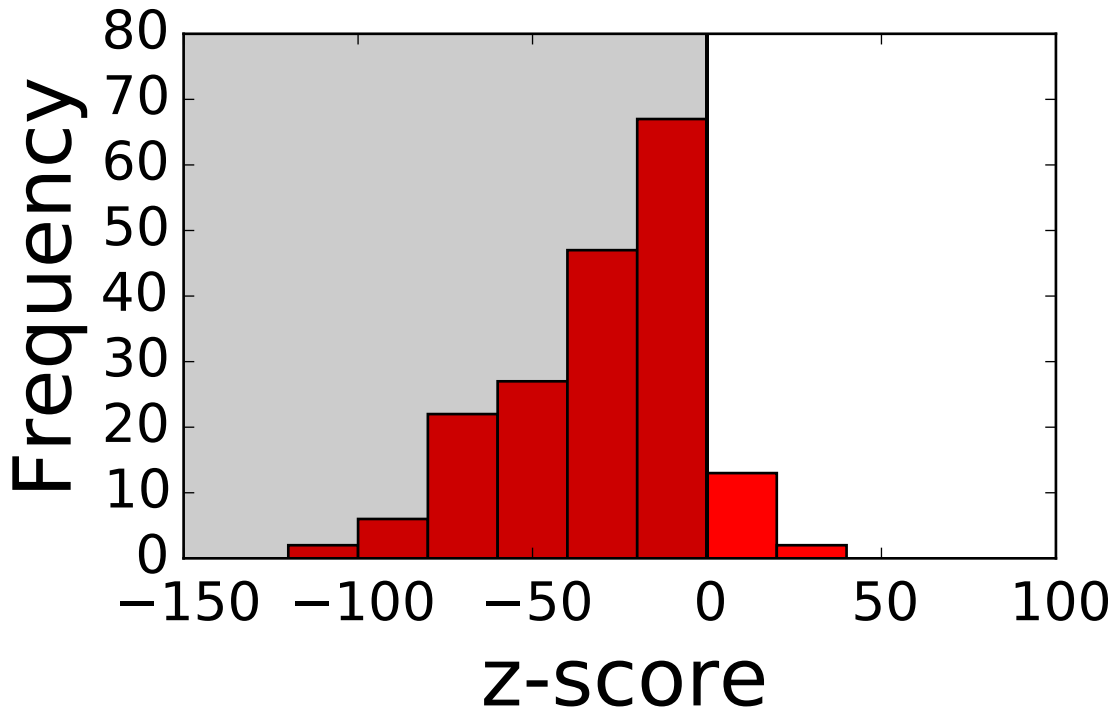

Supplement: Supplemental Material [file supp_g3.116.032227_FileS1.zip › SD_Submission 3/Results/Figures/3B-zscore-histogram-Sgene.pdf]

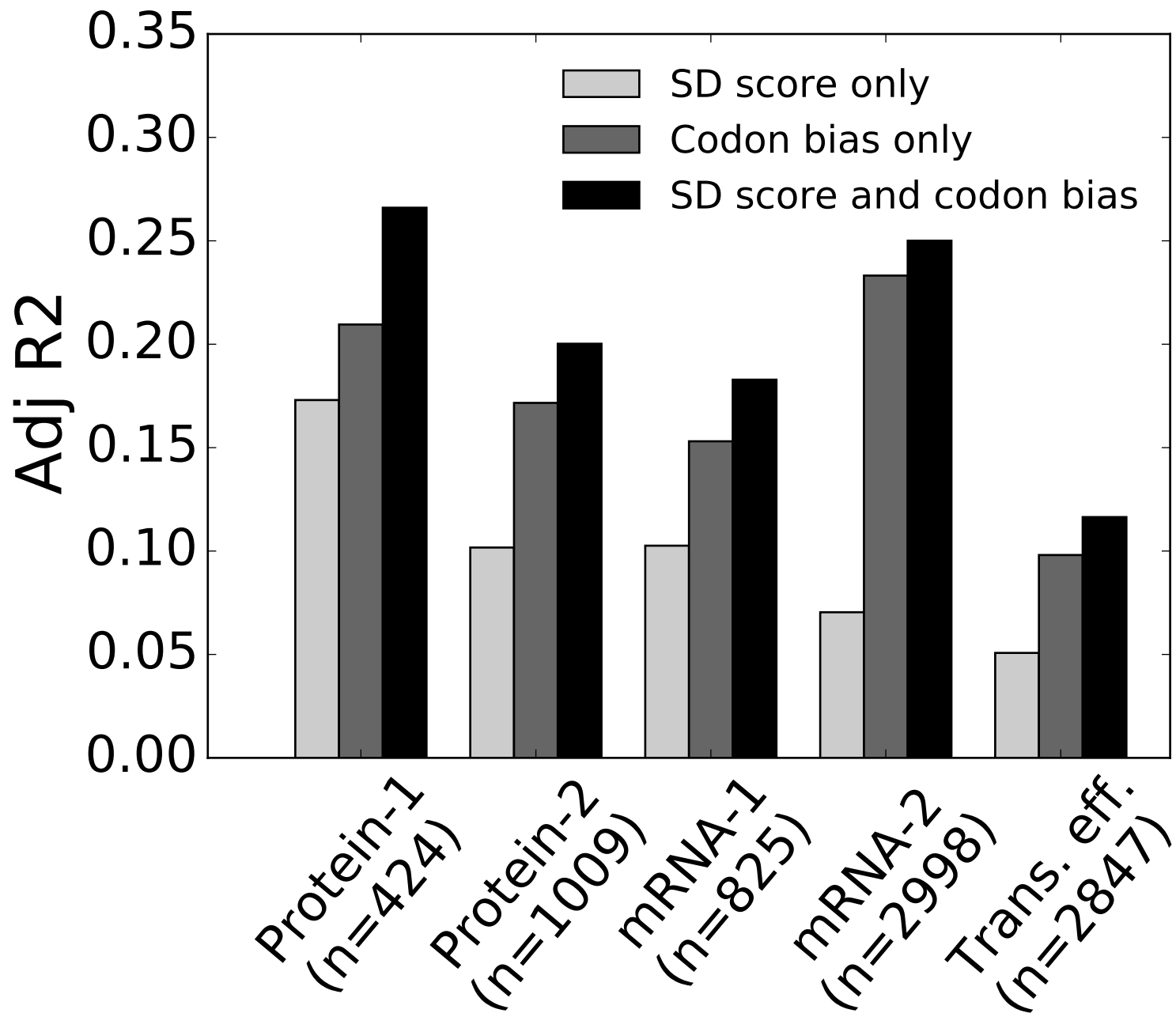

Supplement: Supplemental Material [file supp_g3.116.032227_FileS1.zip › SD_Submission 3/Results/Figures/4B-protein-corr.pdf]

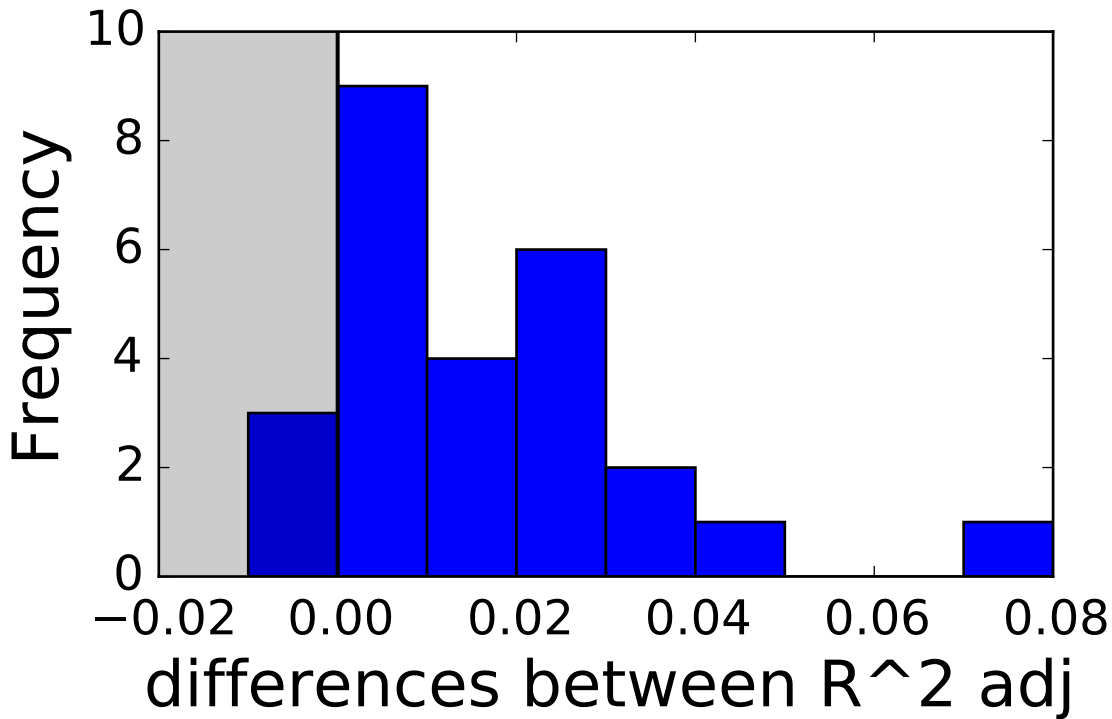

Supplement: Supplemental Material [file supp_g3.116.032227_FileS1.zip › SD_Submission 3/Results/Figures/5-protein-organism-corr-hist.pdf]

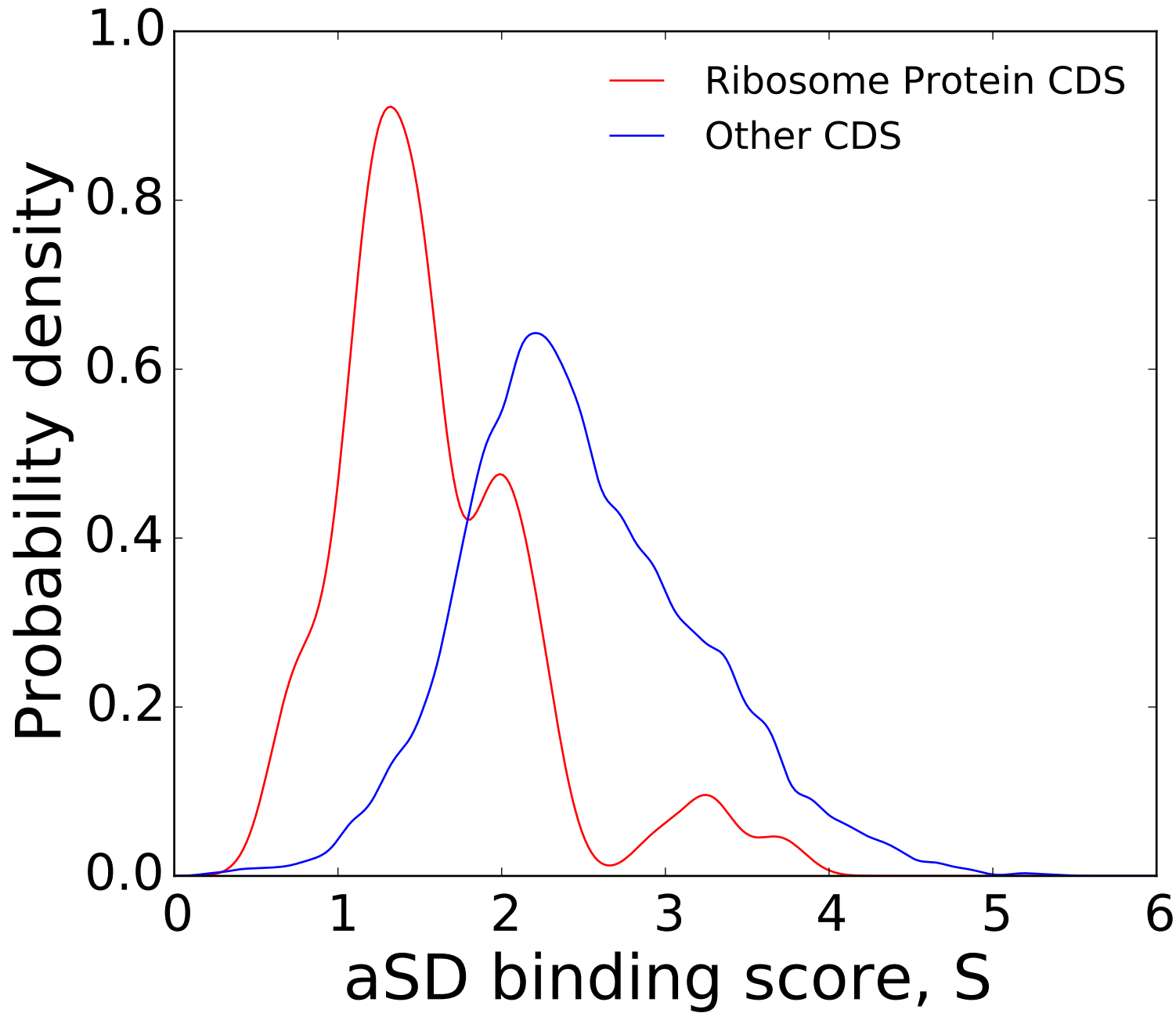

Supplement: Supplemental Material [file supp_g3.116.032227_FileS1.zip › SD_Submission 3/Results/Figures/6A-example-ribosome-subset.pdf]

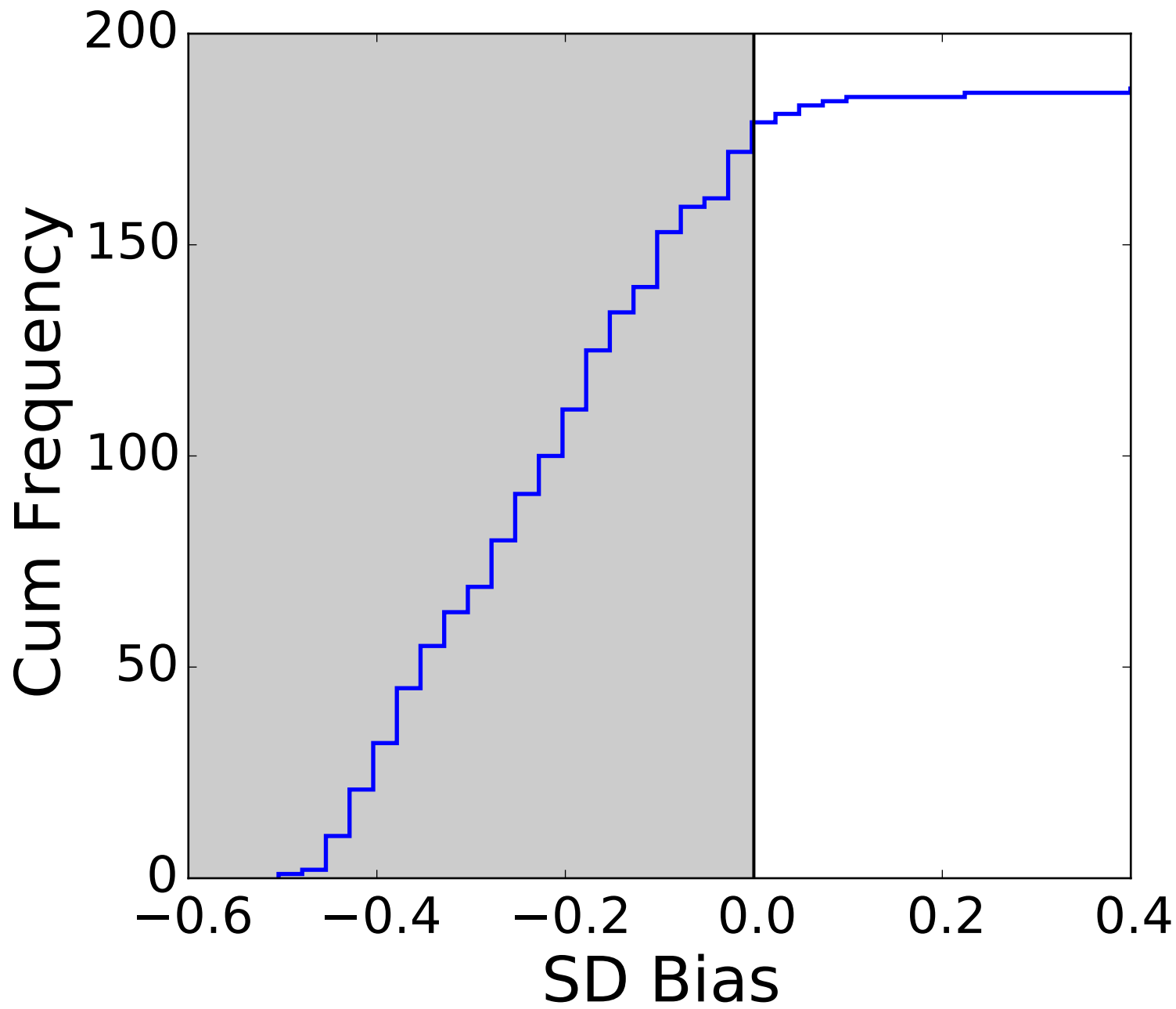

Supplement: Supplemental Material [file supp_g3.116.032227_FileS1.zip › SD_Submission 3/Results/Figures/6B-ribosome-protein-bias.pdf]

Min Doubling  
Time (hr)

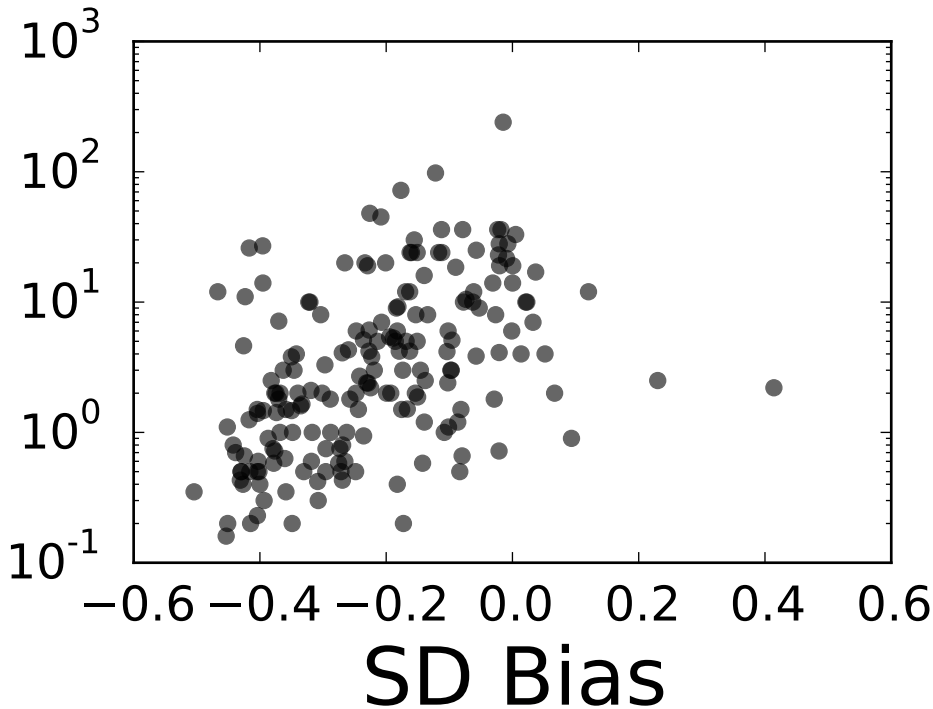

Supplement: Supplemental Material [file supp_g3.116.032227_FileS1.zip › SD_Submission 3/Results/Figures/6C-growth-corr.pdf]

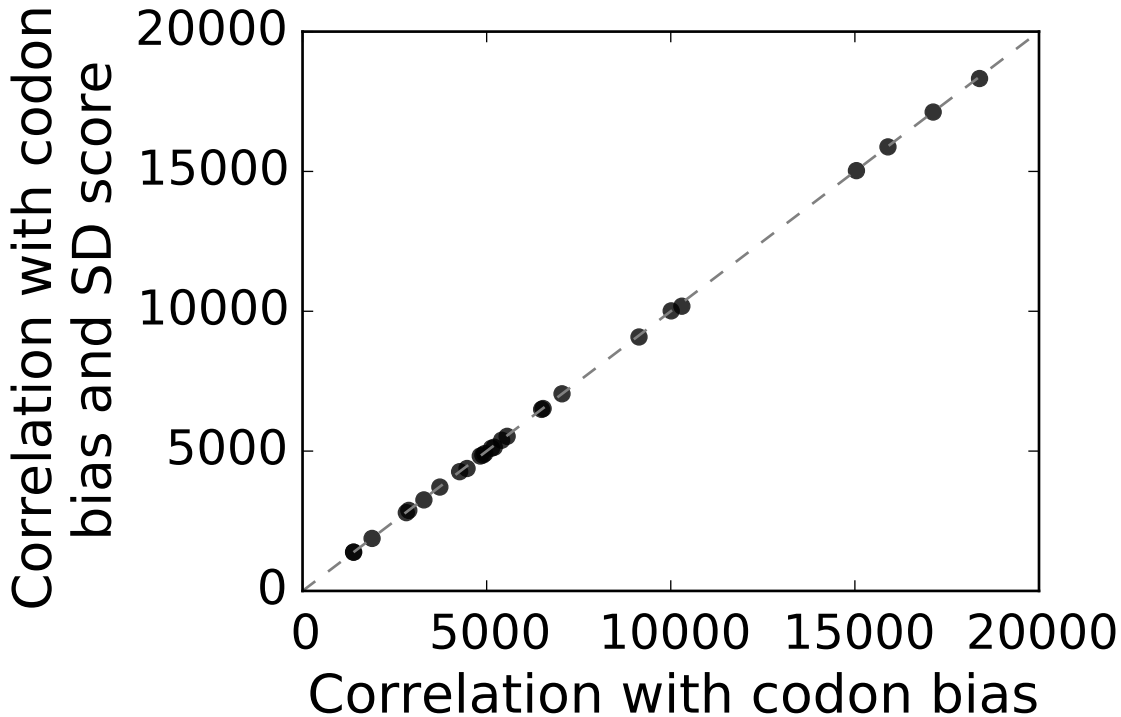

Supplement: Supplemental Material [file supp_g3.116.032227_FileS1.zip › SD_Submission 3/Results/Figures/Sup-protein-organism-corr-aic.pdf]

Correlation with codon  
bias and SD score

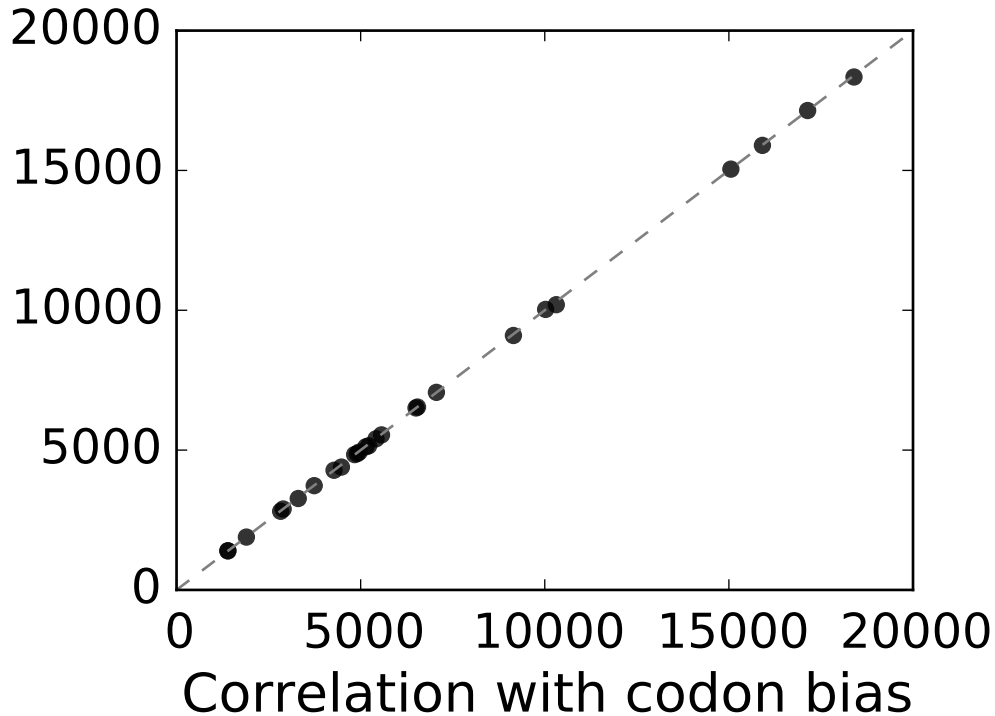

Supplement: Supplemental Material [file supp_g3.116.032227_FileS1.zip › SD_Submission 3/Results/Figures/Sup-protein-organism-corr-bic.pdf]
